# The Mechanism of Allosteric Inhibition of Protein Tyrosine Phosphatase 1B

**DOI:** 10.1371/journal.pone.0097668

**Published:** 2014-05-15

**Authors:** Shuai Li, Jingmiao Zhang, Shaoyong Lu, Wenkang Huang, Lv Geng, Qiancheng Shen, Jian Zhang

**Affiliations:** 1 Department of Pathophysiology, Key Laboratory of Cell Differentiation and Apoptosis of the Chinese Ministry of Education, Shanghai JiaoTong University, School of Medicine (SJTU-SM), Shanghai, China; 2 Medicinal Bioinformatics Center, Shanghai JiaoTong University, School of Medicine, Shanghai, China; Wake Forest University, United States of America

## Abstract

As the prototypical member of the PTP family, protein tyrosine phosphatase 1B (PTP1B) is an attractive target for therapeutic interventions in type 2 diabetes. The extremely conserved catalytic site of PTP1B renders the design of selective PTP1B inhibitors intractable. Although discovered allosteric inhibitors containing a benzofuran sulfonamide scaffold offer fascinating opportunities to overcome selectivity issues, the allosteric inhibitory mechanism of PTP1B has remained elusive. Here, molecular dynamics (MD) simulations, coupled with a dynamic weighted community analysis, were performed to unveil the potential allosteric signal propagation pathway from the allosteric site to the catalytic site in PTP1B. This result revealed that the allosteric inhibitor compound-**3** induces a conformational rearrangement in helix α7, disrupting the triangular interaction among helix α7, helix α3, and loop11. Helix α7 then produces a force, pulling helix α3 outward, and promotes Ser190 to interact with Tyr176. As a result, the deviation of Tyr176 abrogates the hydrophobic interactions with Trp179 and leads to the downward movement of the WPD loop, which forms an H-bond between Asp181 and Glu115. The formation of this H-bond constrains the WPD loop to its open conformation and thus inactivates PTP1B. The discovery of this allosteric mechanism provides an overall view of the regulation of PTP1B, which is an important insight for the design of potent allosteric PTP1B inhibitors.

## Introduction

Protein tyrosine phosphatases (the PTP family), a significant branch of phosphatases, are signaling enzymes responsible for the regulation of multifarious cellular processes, including cell growth, division, adhesion and motility progression throughout the entire life of normal cells [1], [2]. As a superfamily, despite the diversity in size, spatial structure, or intracellular location, PTPs are characterized by a homologous PTP signature motif, (I/V)HCXAGXXR(S/T)G, and a catalytic WPD loop, both of which are highly conserved in the catalytic domain from bacteria to mammals [3], [4].

Protein tyrosine phosphatase 1B (PTP1B) expressed in the human body participates in selective dephosphorylation in various signal transduction pathways [5]. For example, by dephosphorylating the phosphorylated tyrosine of the insulin receptor, PTP1B is able to block the activated insulin receptor pathway, as validated by PTP1B gene deficient mice showing enhanced insulin sensitivity and a decreased incidence of obesity and diabetes [6]. The potential clinical value of the reversible role in the insulin/leptin receptor phosphorylation and signaling provides a major stimulus to the realization that inhibiting PTP1B can alleviate insulin resistance, normalize glycaemic control and address both Type 2 diabetes and obesity [7]–[10]. Thus, the catalytic site with surrounding sub-pockets has been primarily explored through a vast number of investigations to design potential inhibitors [8], [11], [12]. Nevertheless, the highly conserved structural architecture in the active center of PTPs and low bioavailability presents a key challenge in the design and development of selective PTP inhibitors [8]. For instance, PTP1B shares 72% identity overall and 94% identity in the catalytic site residues with T-cell PTPs (TCPTP) [13]. In this context, most competitive inhibitors of PTP1B frequently have lethal adverse effects by affecting the normal function of TCPTP [7], [14].

**Figure 1:**
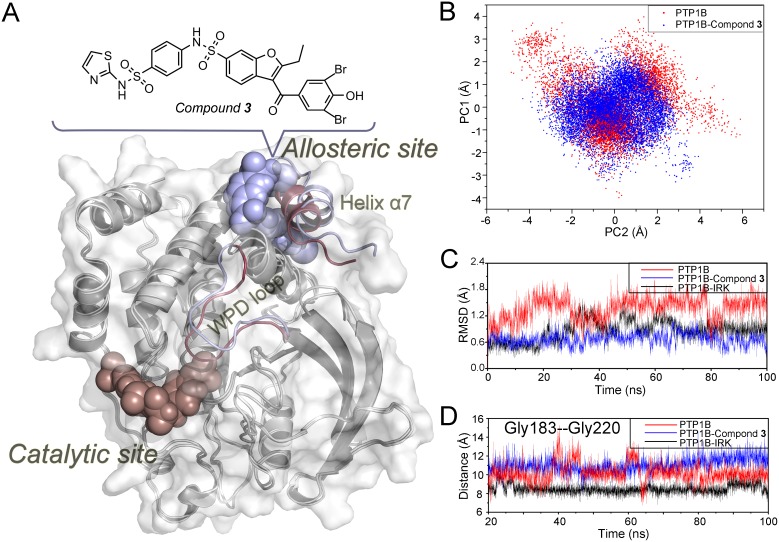
The discrepancy between the average structures of *apo* and compound-3 bound complexes. (A) Comparison of PTP1B in the allosteric inhibitor bound state and *apo* state by superimposing the average structures. The whole structures fit well (shown in silver), excepting certain regions undergoing marked rearrangements (inhibitor bound state–cyan; *apo* state–red). Active sites and allosteric sites are shown as spheres. The allosteric site is located ∼20 Å away from the active site. (B) Projection of the WPD loop on the first two eigenvectors, denoted with PC1 and PC2. Red and blue dots represent samples from the *apo* and compound-**3** bound state MD simulations, respectively. (C) A time evolution of the RMSD was performed on the WPD loop in the *apo* state (red), compound-**3** bound state (blue) and substrate bound state (black) simulations with reference to their respective initial structures. (D) The C_α_ distances between Gly183 and Gly220 among the three MD simulations.

Allosteric sites, because of their lower sequence-conservation pressure compared with evolutionarily conserved catalytic sites, have higher specificity, fewer side effects and lower toxicity and are therefore investigated as a target in drug discovery [15], [16]. To circumvent the bottleneck encountered in the development of PTP1B inhibitors, substantial interest has focused on the design of allosteric PTP1B inhibitors [17], and a druggable allosteric pocket ∼20 Å away from the catalytic site as well as a set of non-pTyr-like allosteric inhibitors were identified (Figure 1) [18]. The novel allosteric site is located on the C-terminal domain of PTP1B and is flanked by helices α3, α6 and α7, which compose a hydrophobic pocket for allosteric inhibitors. Among those chemicals tested, compound-**3** revealed relatively higher potency and selectivity (IC_50_ = 8 µM) over TCPTP [18].

Structurally, the crystal PTP1B-compound**-3** complex shows that the allosteric inhibitor binds to the inactive state of PTP1B with WPD loop (residues 177–185) in its open conformation, which prevents the physiological dephosphorylation reaction [18]. In addition, the displacement and partial uncoiling of helix α7 in the compound-**3** bound PTP1B was observed [18]. Hoff *et al.* suggested that the conserved WPD hydrophobic environment is required in maintaining the normal catalytic activity [19]. Using enzymological based techniques, Picha *et al.* demonstrated that the C-terminal domain has the potential to influence the activity of PTP1B [20]. Through MD simulations and site-directed mutagenesis experiments, several residues located in helices α3, α6, α7 and loop 11 have been respectively determined to play a roles in the regulation of PTP1B function [17], [18], [21]–[26]. Although much knowledge has been gained regarding the relationship between the structure and function of PTP1B, molecular mechanism of allosteric inhibitor from the allosteric site to the catalytic site remains unclear, which may hinder insights into the allosteric regulation of PTP1B and inhibitor design.

In this study, unbiased MD simulations coupled with a dynamic weighted community analysis [27] were performed to identify potential allosteric mechanism in PTP1B. Through a comprehensive analysis of dynamic community network, the allosteric pathway of compound-**3** bound PTP1B, from the allosteric to catalytic sites, was uncovered and key residues involved in the pathway provide novel understanding on the design of potent allosteric PTP1B inhibitors.

## Materials and Methods

### Simulation Systems

The full-length canonical structure of PTP1B is a monomer containing 435 amino acids. The N-terminal domain is the catalytic core domain (residues 1 to 298) and is widely used in computational and biochemical studies [28], [29]. In this study, original structures were selected from the RSC Protein Data Bank (http://www.rcsb.org), and three 100 ns MD simulations were conducted with different states: the *apo* state (PDB code: 2HNP) [29], the allosteric inhibitor bound state (PDB code: 1T4J) [18], and the substrate bound state (PDB code: 1G1H) [6], with the WPD loop presenting in an open, open or closed conformation, respectively. In the allosteric inhibitor bound system, compound-**3** was chosen as the ideal inhibitor model to investigate the allosteric effect because of its potency [18]. The bi-phosphorylated form of the insulin receptor kinase (IRK), a natural substrate of PTP1B, was taken to model the PTP1B-substatre complex in their catalytically competent state to serve as a negative control for the WPD closed conformation [6]. In a previous study, helix α7 was determined to play a pivotal role in the allosteric regulation of PTP1B [24]. However, helix α7 is not represented in the crystal structures of 2HNP and 1T4J. Therefore, helix α7 was determined by superimposing the crystal structure with 1G1H. The initial constructed model was further optimized using the Sybyl6.8 program [30].

### MD Simulations

MD simulations were conducted on these three systems using the program AMBER 11 [31]. The protonation state of ionizable residues was set at the default value for pH 7 except for the C-terminal (–COOH), which was modeled as neutral to avoid artifacts because it is not the actual terminus. The protonation states of the histidine residues were assigned based on the results of a PROPKA calculation (http://propka.ki.ku.dk/) [32]. All His residues were modeled in the neutral state. Whether the HID/HIE state was selected was determined by the local hydrogen bonding network. Previous studies have shown that in physical conditions, Cys215 and Asp181 are present as ionic and protonated forms [33]–[35]. Thus, we changed the PDB files by replacing the residue code CYS and ASP to CYM and ASH to meet the requirements of the amber force field. Then, all of the hydrogen atoms were added to the protein using the Xleap tool from the AMBER suite, and the parameters were assigned according to the AMBER FF03 force field [36]. Partial atomic charges for the non-residue atoms of compound-**3** were calculated using the restricted electrostatic potential fitting protocol implemented in the ANTECHAMBER module of the AMBER 11 program and following electrostatic potential calculations at ab initio HF/6–31G* levels. A truncated octahedral box of TIP3P waters [37] was added with a 10 Å buffer around the complex. Counterions were added to maintain the electroneutrality of these systems.

To remove bad contacts in the initial structures, the steepest descent and conjugate gradient algorithm energy minimization methods were introduced [38], [39]. First, the energy was minimized for water molecules and counterions, with a positional restraint of 500 kcal mol^−1^ Å^−2^ in the complex; the steepest descent method was applied for the first 2,000 steps, and then, the conjugated gradient method was used for the subsequent 3,000 steps. Afterward, the entire system was minimized without any restraints; the steepest descent method was used for the first 4,000 steps, and then, the conjugated gradient method was used for the subsequent 6,000 steps. After minimization, each system was heated gradually from 0 to 300 K within 50 picoseconds (ps). This was followed by a constant temperature equilibration at 300 K for 300 ps, with a positional restraint of 10 kcal mol^−1^ Å^−2^ in the complex for the canonical ensemble (NVT). Finally, a 100 ns MD simulation was performed on these three systems in an isothermal, isobaric ensemble (NPT, T = 300 K and P = 1 atm) with periodic boundary conditions. An integration step of 2 fs was set for the MD simulations, and the long-range electrostatic interactions were treated using the particle mesh Ewald method [40] with a cubic fourth-order B-spline interpolation and by setting the direct sum tolerance to 10^−5^. A cutoff equal to 10 Å was used for short-range electrostatics and van der Waals interactions. The SHAKE method [41] with a tolerance of 10^−5^ Å was applied to constrain all covalent bonds involving hydrogen atoms. The temperature and pressure were coupled with a time constant of 1.0 ps, isotropic position scaling, and a relaxation time of 2.0 ps according to Langevin’s algorithm [42]. Coordinates were saved every 1.0 ps for analysis.

After performing the MD analysis, the C_α_-atoms root mean square deviations (RMSD) of the three MD trajectories were calculated with respect to each initial set of coordinates. As shown in Figure S1, the three systems reached equilibrium after 20 ns with the RMSD values converging approximately to 2.0 Å. Therefore, the trajectories from 20 ns to 100 ns were selected for analysis.

### Principal Component Analysis (PCA)

A principal component analysis (PCA) was performed on the MD trajectories of PTP1B in the *apo* and compound-**3** bound states. Snapshots that were saved every 4 ps (25,000 snapshots) were used to construct a covariance matrix *C*, as in Eq. (1):
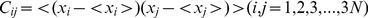
(1)where *x_i_* is a Cartesian coordinate of the *i_th_* C_α_ atom, < *x_i_* > represents the time average over all the configurations selected in the simulation, and *N* is the number of the C_α_ atoms. Prior to the analysis, translation and rotational motions were excluded by overlaying the C_α_ atom of PTP1B to the reference crystal structure.

The diagonalization of *C* generated the eigenvalues, *λ_i_*, and the corresponding eigenvectors, *V_i_*, namely, the principal component (PC). *V_i_* represents the directions in the multidimensional space that correspond to independent modes of atomic motion, whereas *λ_i_* represent their corresponding amplitudes. The first several PCs, ranked according to their *λ_i_*, describe the functionally significant motions in the protein. The projection 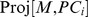 of any structure (snapshot) *M* onto the *i_th_* PC was calculated by Eq. (2):
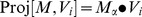
(2)where *M*_α_ is the C_α_ atom of PTP1B after overlaying *M* with the reference crystal structure.

In this study, a PCA was performed to address the collective motions of the WPD loop using the positional covariance matrix, the coordinates of Cα atoms and its eigenvectors in AmberTools. In both states, the first two eigenvectors covered over 50% of the variance, with the cumulated contributions of the first 30_th_ PCs to the conformational changes of the WPD loop shown in Figure S2.

### Dynamic Cross-correlation Matrices

Dynamic cross-correlation matrices (DCCM), as a mutual informatics indicator, are composed of the fluctuations cross-correlations coefficient in the positions of Cα atom during the simulation. The DCCM were calculated according to Eq. (3):
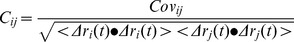
(3)where 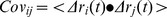 denotes the covariance in motion of the Cα-atoms of residue *i* and *j*; 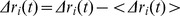. The value of *C_ij_* is between −1 and 1. If *C_ij_* = 1, then the residues are moving in a correlated fashion (same direction) during the simulation, whereas *C_ij_*  =  −1 implies that the residues are moving in an anti-correlated fashion (or in opposite directions). Residues that move independently (or completely uncorrelated) of one another have a correlation value close to 0.

### Allosteric Dynamic Community Analysis

The in-house dynamic community analysis was developed by us based on the method of Rivalta *et al* [43]. In this approach, a network is defined as a set of nodes with connecting edges. The Cα atom of each amino acid is taken as a “node”, and “edges” connect pairs of “nodes” if the corresponding residues are in contact. The combination of these “node” and “edges” build a contact map. Then, the dynamic network map is obtained by weighting those “nodes” in the contact map because every “edge” has a distinct contribution value to the flow of information. Using the Floyd-Warshall algorithm [44], the number of shortest paths that pass through a certain edge, also called “betweeness”, is calculated.

The weighted map is subsequently partitioned into local substructures, “communities”, to reflect the structural units of a protein which directly participates in the allosteric movement. The main rule of dividing “communities” is that “nodes” in a “community” have more and stronger intraconnections than interconnections with “nodes” in other “communities”. Thus, the Girvan-Newmann algorithm [45], which maximizes the modularity measure (*Q*) to ensure the quality of the division strategy, is performed. In particular, the network nodes are connected by edges whose length is weighted by the correlation in motion between the residues:
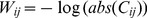
(4)


The correlations coefficients between all residues were analyzed for the 20–100 ns MD simulation frames using the normalized covariance in Eq. (5). If *e_ij_* is the fraction of edges that links nodes in community *i* to nodes in community *j*, then the modularity, *Q*, is defined as:
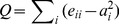
(5)where 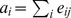 is the fraction of edges that connect to the nodes in community *i*.

In the present study, compound-**3** is not considered as a node, ensuring that the networks for the *apo* and compound-**3** bound PTP1B states have identical number of nodes. Because the communities of the N-terminal PTP1B are distant from the core communities and have no effect on the “allosteric-catalytic sites talk”, the communities of the N-terminal PTP1B were discarded. However, several spatially adjacent and functional similar or non-critical communities underwent a degeneracy process to present more prominent and briefer landscapes with “ball-and-stick” models. After those processes, each community stands for one or several spatially adjacent domains in which the interactions are satisfactorily intensive. For example, C4 stands for the WPD loop and N-terminal domain of the helix α3.

In the ball-and-stick models, each ball stands for an individual community, the volume of which is in direct proportion to the size of the community, that is, the number of residues it contained. The cross sectional-area of each stick linking two balls is positively correlated to the betweeness, which represents the information flow between two communities.

## Results and Discussion

### Conformational Differences for the *apo* and Compound-3 Bound PTP1B States

WPD loop is the primary determinant for the binding of catalytic substrate in PTP1B. MD simulations showed large conformational difference in the intrinsic motion of the WPD loop by the superimposition of the average structures between the *apo* and compound-**3** bound states (Figure 1A). Further analysis by PCA demonstrated that the WPD loop in the *apo* PTP1B underwent more diverse conformations than that in the compound-**3** bound PTP1B (Figure 1B), indicating the inherent flexibility of the WPD loop in the *apo* state. The difference was also observed in the RMSD calculation of C_α_ atoms (Figure 1C), the WPD loop randomly fluctuated in the *apo* state but maintained its open conformation in compound-**3** bound state throughout the MD simulations. To quantitatively investigate the changes of the WPD loop, the time-dependent distance between Gly183 located in the top of the WPD loop and Gly220 located in the catalytic P loop (residues 214–221) were measured. As shown in Figure 1D, substrate (IRK) stabilized the active closed conformation of the WPD loop at the distance of ∼8.5 Å and compound-**3** kept the open conformation of the WPD loop at the distance of ∼12 Å. However, the distance in the *apo* PTP1B fluctuated between 8 and 13 Å. This result suggested that the WPD loop is able to switch between ‘open’ and ‘closed’ conformations in physiological conditions, with substrate and allosteric ligands exerting distinct effects on the ‘open-closed’ switch of the WPD loop of PTP1B. Furthermore, the relationship between the WPD loop and allosteric compound-**3** was preliminarily obtained from the dynamic conformations of the WPD loop in these systems. PTP1B is intrinsically dynamic in its *apo* state, with the WPD loop uncommitted in its flexibility and conformation. After the allosteric compound-**3** is bound, the WPD loop is committed in its open conformation and is incapable of participating in normal physiological reactions.

In addition to the WPD loop, MD simulations showed that helix α7 in the allosteric site underwent a conformational rearrangement when compound-**3** binds to PTP1B, which is in good agreement with previous biophysical changes detected by the fluorescent labeling method [46]. This observation also suggested that the motion of helix α7 could be coupled to the intrinsic motion of the WPD loop. To further illuminate the allosteric pathway of PTP1B from the allosteric site to the WPD loop (catalytic site) after compound-**3** is bound, a dynamic community analysis, coupled with the analysis of the MD trajectories, was used to unravel the potential allosteric mechanism.

### Allosteric Pathway Analysis by Dynamic Communities

**Figure 2:**
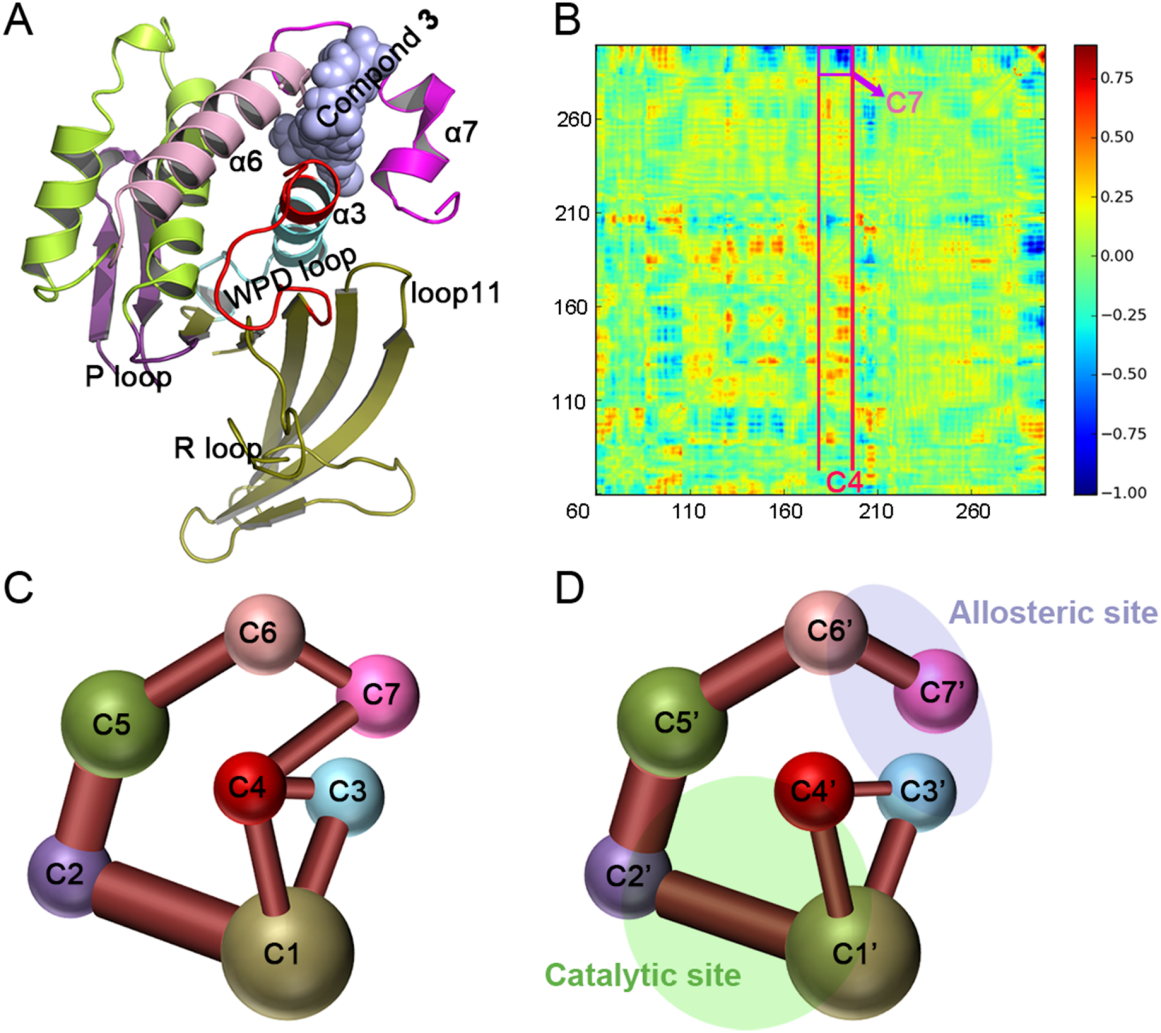
Dynamic community analysis of both the *apo* and compound-3 bound PTP1B. (A) Structural domains matching up to their communities are highlighted in consistent colors. Notable structural elements are also shown on the protein structure. (B) Visualization of the correlation coefficient matrix based on the MD trajectories for the *compound-* ***3*** *-minus-apo* PTP1B complexes. Note, dramatic changes have taken place in the correction of the magenta box between C7 (helix α7) and C4. (C) and (D). Color coded optimal community network of the *apo* and compound-**3** bound state with ball-and-stick models. Each ball stands for an individual community. The stick stands for the “betweeness”. Color scheme: C1, C1’(gold); C2, C2’(purple); C3, C3’(cyan); C4, C4’(red); C5, C5’(green); C6, C6’(pink); and C7, C7’(magenta). C3 (C3’) stands for the C-node of helix α3. C4 (C4’) stands for the WPD loop and N-terminal helix α3. C7 stands for helix α7. The partial region of C1’ (C1), C2 (C2’) and C4’ (C4) form the active site. The allosteric site consists of parts of C3’ (C3), C6’ (C6) and C7’ (C7).

The dynamic community analysis was performed on both *apo* and compound-**3** bound PTP1B (see Materials and Methods), the optimal community networks were presented in Figure 2 and residues attributed to each community were listed in the Table S1-S2. In the analysis, the community structures typically reveal communities of nearby residues with common secondary structure elements, although they may be distant in sequence [27]. To clarify the community networks, structural domains corresponding to their communities were illustrated with consistent colors (Figure 2A, 2C and 2D). In the community network, C4 (C4’) is an important community located at the center and strongly connected to the surrounding communities. This community represents the local environment of the WPD loop, containing the whole WPD loop and part of the N-terminal helix α3. C7 (C7’) is composed of residues in helix α7. From the dynamic community analysis, the betweeness connecting C7 and C4 in the *apo* state diminished after compound-**3** is bound, whereas most of the communities and their betweeness changed subtle, which is consistent with the conformational superimposition between the *apo* and compound-**3** bound PTP1B (Figure 1A). This finding indicated that the information exchange between helix α7 and C4 community in the allosteric compound-**3** bound state disappeared, with helix α7 departing from the core protein. The betweeness connecting helix α7 with C4 community includes the interactions from the hydrophobic WPD loop environment [19] as well as the triangular interactional region composed of the N-terminal helix α3, helix α7 and loop 11 [24]. Thus, these interactions could contain an allosteric pathway from helix α7 in the allosteric site to the WPD loop in the catalytic site. In addition, the differential dynamic cross correlation matrix (DCCM) between the *apo* and compound-**3** bound PTP1B trajectories also supported that the most dramatic changes occurred in the joint between C7 and C4 communities when compound-**3** binds to the allosteric site of PTP1B (Figure 2B).

### Allosteric Pathway Analysis by Key Residues

**Figure 3:**
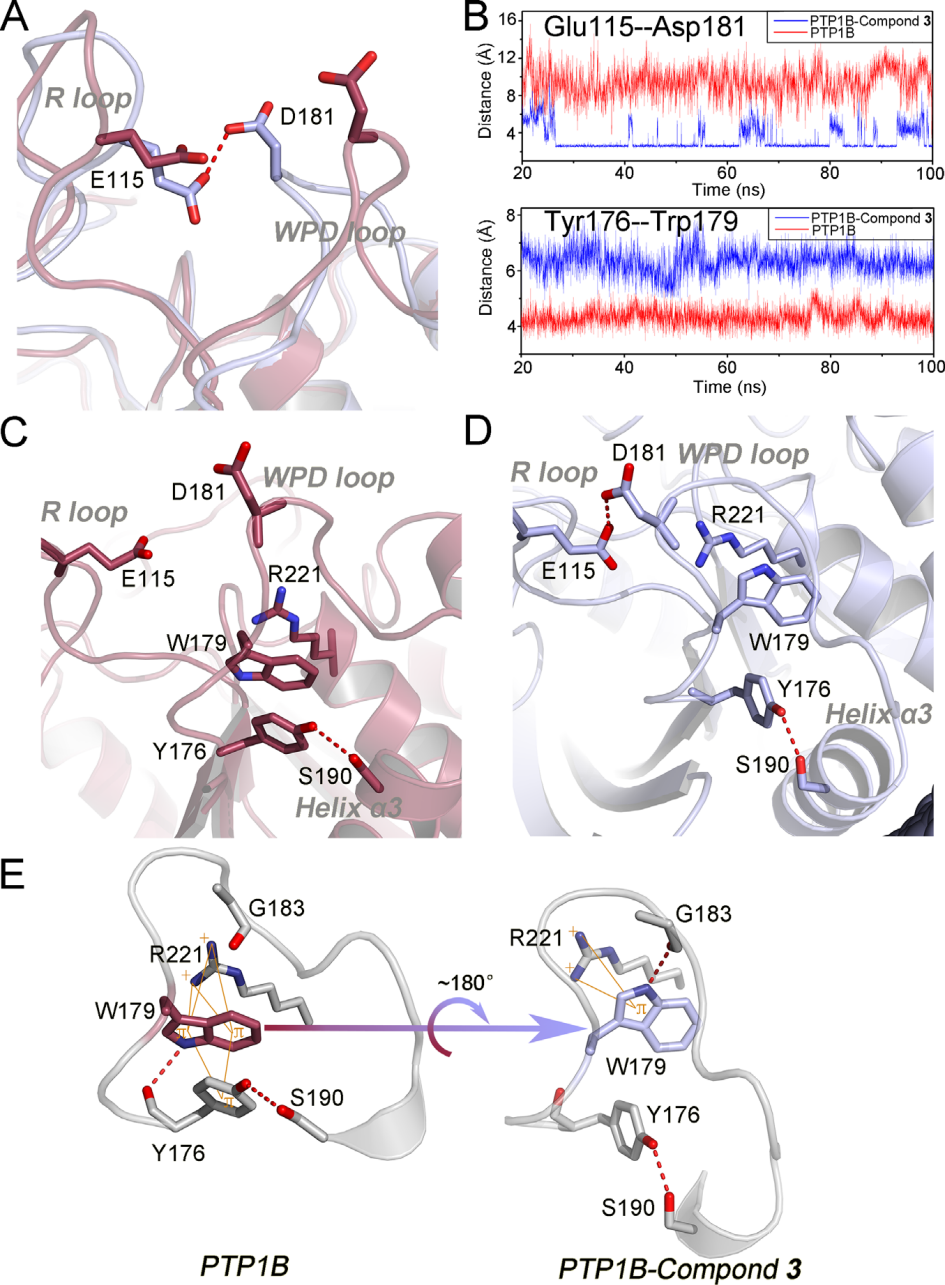
Conformational rearrangements in the conservative WPD domain for both states. (A) Comparison of the region between the WPD loop and R loop in the *apo* state (red) and compound-**3** bound state (cyan). (B) The distance between the OE atom in Glu115 and OD atom in Asp181 (top) and the distance of the side chains between Trp179 and Arg221 (bottom) were monitored in the *apo* state (red) and compound-**3** bound state (blue). (C) and (D)View of the hydrophobic environment of the WPD loop in the *apo* state (red) and the compound-**3** bound state (cyan). (E) View of the indole ring of Trp179 in the *apo* state (red) and the compound-**3** bound state (cyan).

The community analysis showed that the binding of compound-**3** altered the community network of the *apo* PTP1B and the interactions between C7 (helix α7) and C4 communities are crucial for the signal to propagate from the allosteric to the catalytic sites. To elaborate the allosteric pathway at the atomic level, a comprehensive structural analysis of the MD trajectories was performed. The above results suggested that the binding of the allosteric compound-**3** to PTP1B inhibited its catalytic activity by restricting the movement of the WPD loop in the open conformation. Structural analysis showed that this effect was caused by the formation of an H-bond between Asp181 and Glu115 (Figure 3A), which fixed the WPD loop to the R loop of PTP1B when compound-**3** is bound. Furthermore, the time-dependent distance between Glu115 and Asp181 was monitored in both *apo* and compound-**3** bound PTP1B. As shown in Figure 3B, the averages distance of ∼2.5 Å in the compound-**3** bound PTP1B indicated a stable mutual interaction throughout the simulation, which was confirmed by ∼72% occupancy of the H-bond in the compound-**3** bound state. Conversely, the average distance of 10±2.5 Å in the *apo* PTP1B revealed the unconstrained WPD loop when the H-bond did not exist.

Biochemical experiments demonstrated that the dephosphorylation reaction of PTP1B requires the WPD loop lid over its substrate to form a catalytically competent complex [47]–[51]. Our results showed that the binding of allosteric compound-**3** to PTP1B induced the new H-bond between Glu115 and Asp181 to couple the WPD loop to the R loop and resulted in the WPD loop consistently maintaining an open conformation, which eradicated the normal dephosphorylation reaction. Further residue analysis from the dynamic communities (Table S1–S2) indicated that the formation of H-bond was derived from interaction changes propagated from the triangular interactional region of the N-terminal helix α3, helix α7 and loop 11 to the hydrophobic WPD loop environment, which is composed of three conserved residues Tyr176, Trp179 and Arg221.

Mutations found in *Yersinia* PTPase revealed that the corresponding residues of Tyr176, Trp179 and Arg221 could be involved in the allosteric regulation of catalysis [19]. Here, in the trajectory of *apo* PTP1B, the side chain of Trp179 was parallel to both Arg221 by hydrophobic interaction and Tyr176 by π-π interaction throughout the whole MD simulation (Figure 3C). However, the π-π coupling between Trp179 and Tyr176 was broken and the indole ring in Trp179 rotated nearly 180° due to the pulling of Tyr176 when compound-**3** binds to PTP1B (Figure 3D). In Figure 3B, the difference between the *apo* and compound-**3** bound states was shown by the time-dependent distance between Trp179 and Tyr176. As the result of compound-**3** binding, Tyr176 was pulled outward to enhance the interaction with Ser190 in the N-terminal helix α3 of the triangular interactional region and then Trp179 was engaged in a new H-bond with Gly183, the changes of these intrinsic interactions initiated the downward movement of the WPD loop to form the H-bond between Glu115 and Asp181 (Figure 3D–3E) and also supported the regulation of allosteric communication through Tyr176, Trp179 and Arg221 in human PTP1B.

**Figure 4:**
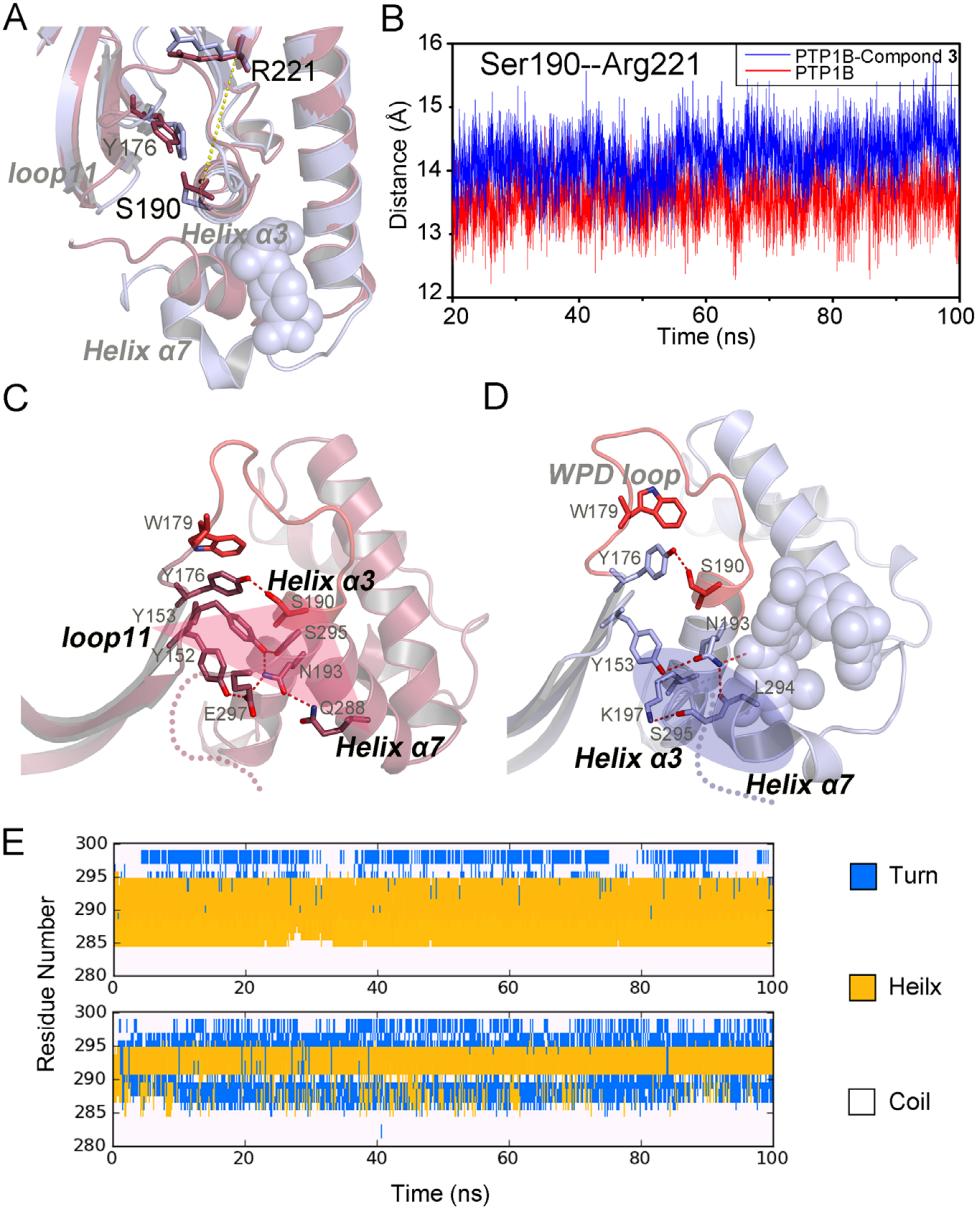
Structural reconstruction of the dynamic interactions for helix α7 with helix α3. (A) Comparison of the distance between Arg221 and Ser190 in the *apo* (red) and compound-**3** bound state (cyan). (B) Tracing the distance between Ser190 and Arg221 in in the *apo* (red) and the compound-**3** bound state (blue). (C) and (D) View of the triangular interactional region among N-terminal helix α3, helix α7, loop 11 in the *apo* (red) and the new interactional interfaces between helix α7 and helix α3 after compound-**3** bound (cyan). (E) The results of Define Secondary Structure of Proteins (DSSP) [52] derived from the PTRAJ module show that the N-terminal helix α7 (residue 285–291) uncoils in the compound-**3** bound state (bottom).

Our results showed that the conformational changes of the hydrophobic WPD loop environment was triggered by the pulling from Ser190 in the N-terminal helix α3 to Tyr176 when compound-**3** binds to PTP1B. The outward movement of helix α3 was further confirmed by the distances between Arg221 and Ser190, which increased by ∼1 Å in the compound-**3** bound state compared to the *apo* state (Figure 4A, B).

Previous studies showed the N-terminal helix α3, helix α7 and loop 11 forms the stable triangular interactional region in the unbound state by hydrogen bonds and polar interactions [17]–[25]. In our simulation of the *apo* PTP1B, the three components maintained the triangular frame without allosteric site (Figure 4C), where the N-terminal helix α3 firmly coupled with loop 11 and helix α7 by the dynamical interactions among Tyr152, Tyr153, Ser190, Asn193, Gln288, Ser295 and Glu297. Olmez *et al* suggested that the stabilization of this structural feature through intensive interactions was closely associated with the physiological conformation of the WPD loop in *yersina* PTP and PTPL1 [24], and the missing of helix α7 in truncated PTP1B in modeling proposed the significant reduction in the flexibility of the catalytic WPD loop [24]. Consistent with above proposals, our MD simulations showed that upon the binding of compound-**3**, the intrinsic interactions among the triangular interactional region were disrupted and rearranged. To accommodate compound-**3**, accompanied with the partial uncoiling of the N-terminal helix α7 (residue 285–291), the remaining C-terminal helix α7 swung outward and reconstructed the interactions with the C-terminal helix α3, resulting in the outward movement of helix α3 and following conformational changes on the hydrophobic WPD loop environment (Figure 4D), as the description of diminished betweeness between C4 and C7 in the community analysis (Figure 2C and 2D). The uncoiling changes of helix α7 were also revealed by a Define Secondary Structure of Proteins (DSSP) analysis in Figure 4E [52]. Finally, the allosteric propagation from helix α7 induced the constraint of WPD loop through the triangular interactional region and the hydrophobic WPD loop environment when compound-**3** binds to PTP1B.

## Conclusions

**Figure 5:**
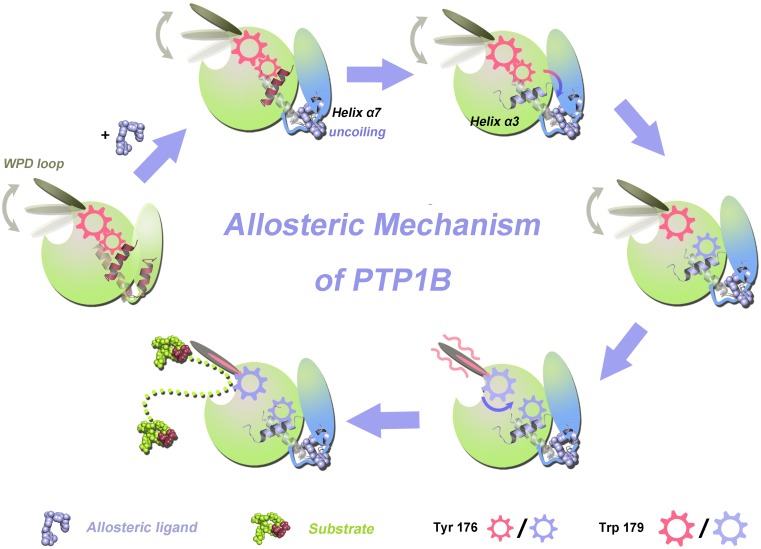
The allosteric signal pathway for PTP1B propagating from the allosteric to the active site.

In this study, MD simulations were performed to elucidate the allosteric inhibitory mechanism of PTP1B (Figure 5). Upon binding of the allosteric compound-**3** to PTP1B, helix α7 uncoiled and was displaced to accommodate this ligand. The resulting conformational rearrangements of helix α7 disrupt the triangular interaction among helix α7, helix α3, and loop11. Helix α7 provides a force to pull helix α3 outward, which enables Ser190 to drag Tyr176 outward. This outcome leads to the outward movement of Tyr176, thereby abrogating the hydrophobic interactions with Trp179 in the WPD loop. The deletion of the hydrophobic interactions between Tyr176 and Trp179 results in a near 180° flip for Trp179. As a consequence, the rotation of Trp179 causes the downward movement of the WPD loop, forming an H-bond between Asp181 and Glu115. The formation of this H-bond couples the WPD loop to the R loop and consequently constrains the WPD loop in its open conformation. The open conformation of the WPD loop is unable to engage in the dephosphorylation reaction, thereby eliminating its catalytic activity. As the key structural features along the allosteric pathway are highly conserved in the PTP family, the PTP1B allosteric pathway may provide insights for other enzymes in the PTP family and contribute to the next generation of PTP1B allosteric drug discovery [53]–[59].

## Supporting Information

Figure S1 Time evolution of the RMSD of the three MD trajectories were calculated in the apo state (red), the compound-3 bound state (blue) and substrate bound state (black) simulations with reference to their respective initial structures.
(TIF)

Figure S2 Cumulated contributions of the first 30th PCs for the conformational changes of the WPD loop.
(TIF)

Table S1 The protein dynamical weighted communities after degeneracy process (shown as Figure 2C) and the residues they contained in the apo PTP1B system.
(DOC)

Table S2 The protein dynamical weighted communities after degeneracy process (shown as Figure 2D) and the residues they contained in the compound-3 bound PTP1B system.
(DOC)
