# Supplementary figures and images for: The Mechanism of Allosteric Inhibition of Protein Tyrosine Phosphatase 1B
Source: PLoS One. 2014 May 15;9(5):e97668. doi: 10.1371/journal.pone.0097668 (PMC4022711; doi:10.1371/journal.pone.0097668)

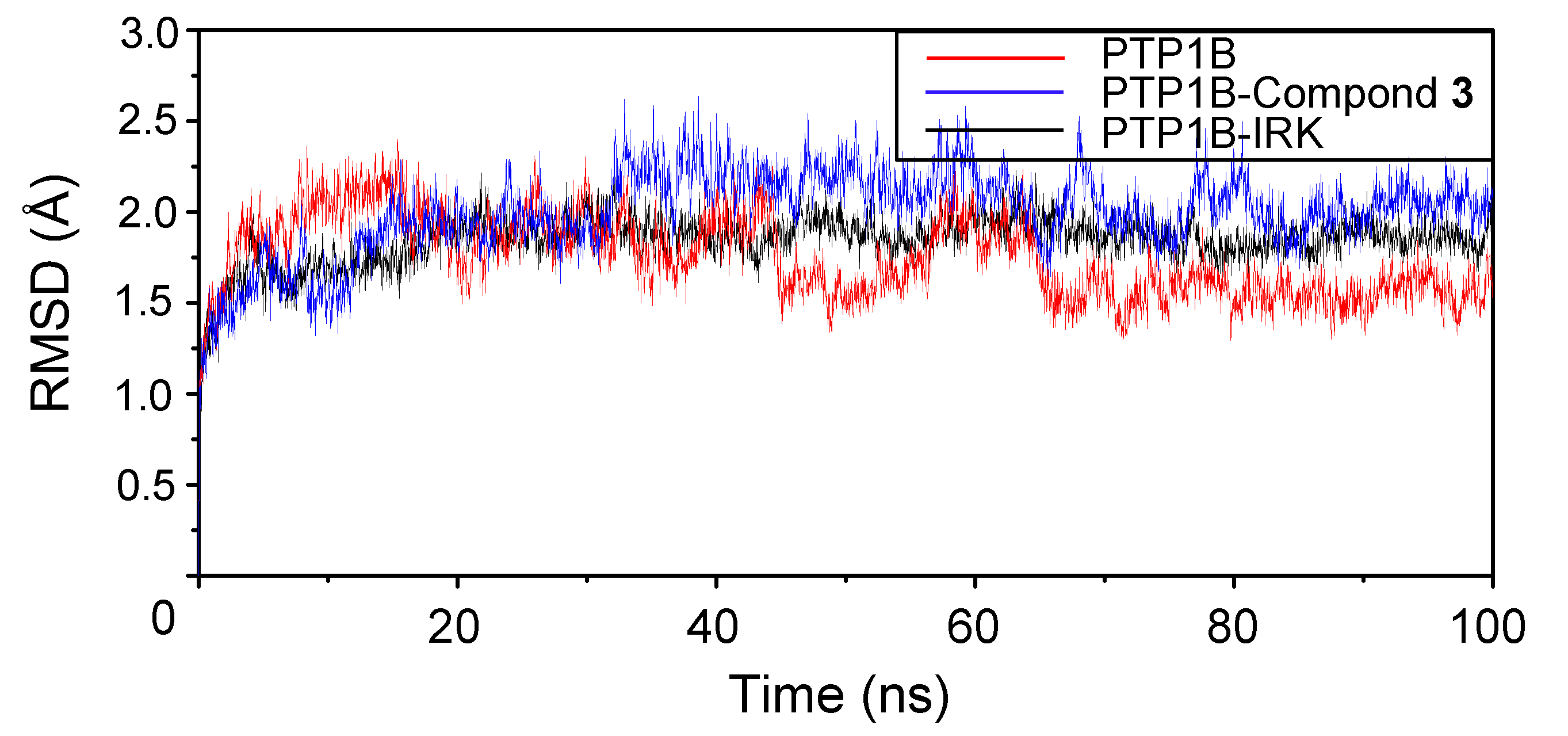

Supplement: Figure S1 — Time evolution of the RMSD of the three MD trajectories were calculated in the apo state (red), the compound-3 bound state (blue) and substrate bound state (black) simulations with reference to their respective initial structures. (TIF) [file pone.0097668.s001.tif]

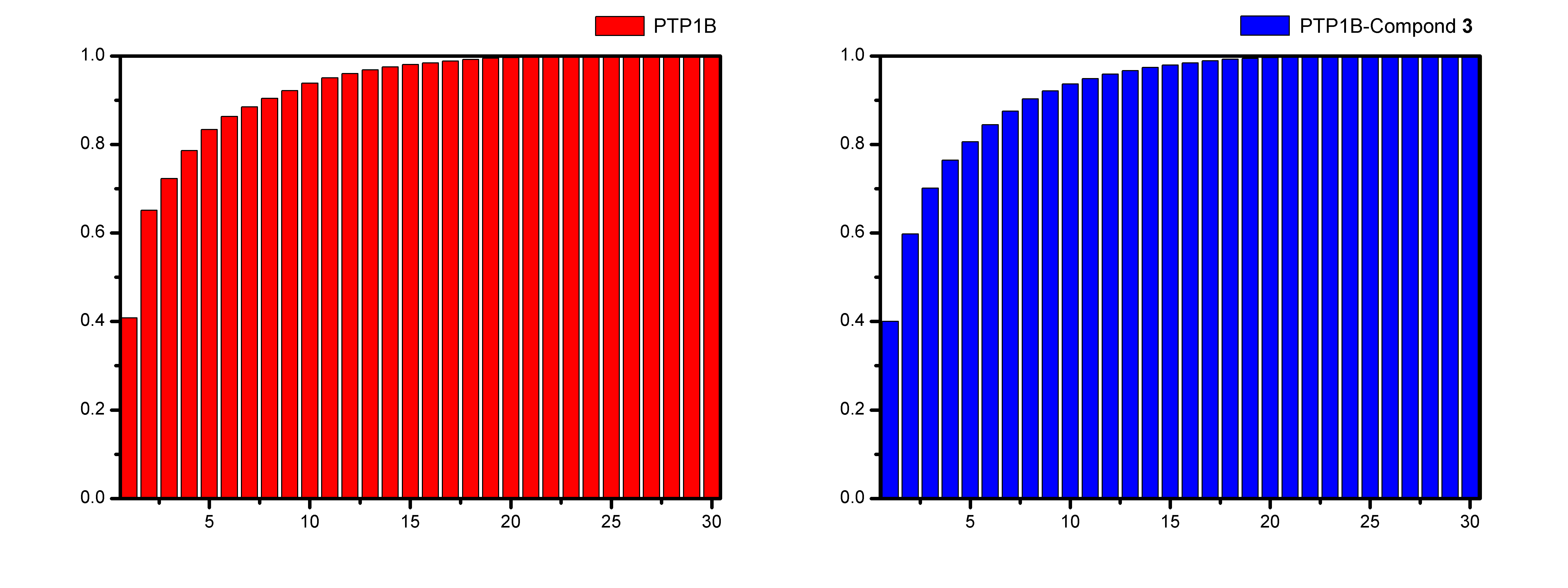

Supplement: Figure S2 — Cumulated contributions of the first 30th PCs for the conformational changes of the WPD loop. (TIF) [file pone.0097668.s002.tif]
