# Supplementary material for: The Mechanism of Allosteric Inhibition of Protein Tyrosine Phosphatase 1B
Source: PLoS One. 2014 May 15;9(5):e97668. doi: 10.1371/journal.pone.0097668 (PMC4022711; doi:10.1371/journal.pone.0097668)
Supplement: Table S1 — The protein dynamical weighted communities after degeneracy process (shown as Figure 2C) and the residues they contained in the apo PTP1B system. (DOC) [file pone.0097668.s003.doc]

**Table S1.** The protein dynamical weighted communities after degeneracy process (shown as Figure 2C) and the residues they contained in the *apo* PTP1B system.

| **Community ID** | **Residue ID** |
| --- | --- |
| **C1** | 104 105 106 107 108 109 110 111 112 113 114 115 116 117 118 119 120 121 122 123 124 125 126 127 128 129 130 131 132 133 134 135 136 137 138 139 140 141 142 143 144 145 146 147 148 149 150 151 152 153 154 155 156 157 158 159 160 161 162 163 164 165 166 167 168 169 170 171 172 173 174 175 176 177 178 209 210 211 212 213 |
| **C2** | 69 70 71 72 73 74 75 76 77 78 79 80 81 82 83 84 85 214 215 216 217 218 |
| **C3** | 192 193 194 195 196 197 198 199 200 201 202 203 204 205 206 207 208 |
| **C4** | 179 180 181 182 183 184 185 186 187 188 189 190 191 |
| **C5** | 219 220 221 222 223 224 225 226 227 228 229 230 231 232 233 234 235 236 237 238 239 260 261240 241 242 243 244 245 246 247 248 249 250 251 252 253 254 255 256 257 258 259 |
| **C6** | 262 263 264 265 266 267 268 269 270 271 272 273 274 275 276 277 278 279 |
| **C7** | 280 281 282 283 284 285 286 287 288 289 290 291 292 293 294 295 296 297 298 299 |
